# What Neuroscientists Think, and Don’t Think, About Consciousness

**DOI:** 10.3389/fnhum.2022.767612

**Published:** 2022-02-24

**Authors:** Peter D. Kitchener, Colin G. Hales

**Affiliations:** Department of Anatomy and Physiology, University of Melbourne, Parkville, MO, Australia

**Keywords:** consciousness, electromagnetism, information, neural signals, computation

## Abstract

The approach the majority of neuroscientists take to the question of how consciousness is generated, it is probably fair to say, is to ignore it. Although there are active research programs looking at correlates of consciousness, and explorations of informational properties of what might be relevant neural ensembles, the tacitly implied mechanism of consciousness in these approaches is that it somehow just happens. This reliance on a “magical emergence” of consciousness does not address the “objectively unreasonable” proposition that elements that have no attributes or properties that can be said to relate to consciousness somehow aggregate to produce it. Neuroscience has furnished evidence that neurons are fundamental to consciousness; at the fine and gross scale, aspects of our conscious experience depend on specific patterns of neural activity – in some way, the connectivity of neurons computes the features of our experience. So how do we get from knowing that some specific configurations of cells produce consciousness to understanding why this would be the case? Behind the voltages and currents electrophysiologists measure is a staggeringly complex system of electromagnetic fields – these are the fundamental physics of neurons and glia in the brain. The brain is entirely made of electromagnetism (EM) phenomena from the level of the atoms up. The EM field literally manifests the computations, or signaling, or information processing/activities performed by connected cellular ensembles that generate a 1st-person perspective. An investigation into the EM field at the cellular scale provides the possibility of identifying the outward signs of a mechanism in fundamental terms (physics), as opposed to merely describing the correlates of our mental abstractions of it.

*To the theoretical question, Can you design a machine to do whatever a brain can do? The answer is this: If you will specify in a finite and unambiguous way what you think a brain does do with information, then we can design a machine to do it. Pitts and I have proven this construction. But can you say what you think brains do?*

McCulloch (1965)

## Neuroscientists and the Neuroscience of Consciousness

Neuroscience research, led by the funding that supports it, is dominated by research into disorders of the nervous system. The pursuit of treatments and cures (and the research efforts into understanding the normal function of the brain) builds on decades of discovery into all levels of neural organization – seemingly none of it reliant on knowing very much at all about consciousness. Studies of how consciousness is generated, and why it has the characteristics it does, is nevertheless a focus of considerable interest and effort. There is no consensus about how it is generated, or how best to approach the question, but all investigations start with the incontrovertible premise that consciousness comes about from the action of the brain.

A key driver of a general understanding of how the nervous system works are the discoveries relating to how signals are combined and transmitted by neurons. From Golgi’s and Ramon-y-Cajal’s insights that neurons are morphologically specialized to form extensive interconnectivity (Glickstein, 2006), a sample of Nobel prizes provides an effective summary of the progress in our understanding of how neurons perform the functions of the nervous system: to Eccles, Hodgkin, and Huxley for “discoveries concerning the ionic mechanisms involved in excitation and inhibition in the peripheral and central portions of the nerve cell membrane”; to Katz, von Euler, and Axelrod for “discoveries concerning the humoral transmitters in the nerve terminals and the mechanism for their storage, release and inactivation,” and to Neher and Sakmann for “discoveries concerning the function of single ion channels in cells.” These discoveries were crucial for understanding how neurons generate, transmit and integrate biological signals and have had an understandably huge impact across all of neuroscience (Eccles, 1982; Valenstein, 2002; Augustine and Kasai, 2007; Reyes, 2019). Central to this understanding is the deep insight of how the biophysics of ionic movement across the neuronal membrane expresses the action potential. The significance and influence of this discovery is captured admirably in McComas’ history “Galvani’s Spark”:

*“The nerve impulse is the mechanism by which the brain conducts its affairs, the currency for all its transactions”* McComas (2011)

Another immensely significant and influential discovery points directly to how the action of neuronal circuits identifies features in the visual scene that form elements of visual perception. By mapping the adequate stimuli of receptive fields in the visual system, Hubel and Wiesel [see Constantine-Paton (2008) and Wurtz (2009) for review] discovered that the connectivity between neurons along the pathway dictates that the receptive fields in visual cortex are tuned to features such as edges and boundaries. Neuronal receptive fields are a product of the inputs they receive so it seems very reasonable to consider that the neurons of the visual pathway, by virtue of their signaling configuration, compute (in some sense) features of the perceptual experience.

In its generalized recent form, what has become “computation by synaptic connectivity” is accepted as the basis of nervous system function. This has convergent support from formal computing. In recent times, digital computers implementing artificial neural networks show that the simple learning rules that define an optimization process cause (for example) convolution kernels to converge from an initial random configuration to a collection of filters that are optimally activated by various oriented edges and simple luminance distributions that comprise the features of the images (Linsker, 1986a,b,c; Olshausen and Field, 1996; Gregor and LeCun, 2010). Deeper layers of these networks become maximally activated by more abstract features of images. These properties, as well as the reliance of learning (network adaptation) through interaction with stimuli, and the lack of explicit specified initial connectivity (and their eventual accuracy), seem to embody many attributes of biological visual systems. This understanding of the brain has become a powerful driver of modern progress in artificial intelligence (LeCun et al., 2015; O’Shea and Nash, 2015; Schmidhuber, 2015).

It is probably difficult to overstate the influence that the rapid development and exploration of formal computation (and especially digital computing) has had on our ideas about how the brain works. Rather than taking formal computation as a metaphor, it is not at all uncommon to hear present day brain scientists ask not *whether* the brain is any sort of computer but rather, *what sort* of computer is it.^1^ For example, the recent enterprise of connectomics assumes not only that the computational aspects of the brain are sufficiently represented in the connectome, but also that the revealed connectome will provide the best possibility of answering questions about how the brain works. The claim that “*Neuroscience would be much easier if we had a detailed circuit diagram of the brain*” (Martin, 2006) may be true, but it is not so clear that the connectome’s explanatory power would extend to how the brain generates consciousness.

What is interesting is that a scientific account of consciousness need form no explicit part of what motivated the cited progress in neuroscience. Indeed, attention to consciousness has woven a history of considerable scientific-cultural controversy into it. Neuroscience is a relative late comer to the question of how consciousness arises, and it is only in the last decades that this topic obtained a level of legitimacy within neuroscience research. This transformation can arguably be specifically dated to the 1990 work of Francis Crick (another Nobel Laureate) and Kristoff Koch that gave birth to the (neurobiological) “correlates-of-consciousness” empirical paradigm (Crick and Koch, 1990) and its descendants. By organizing the science around isolation of observational “correlates-of” consciousness, a physical science could finally get permission to deal with consciousness without its related funding application being tainted by a historically “career-limiting” direct attack on what was then a taboo explanandum in the physical sciences: the 1st-person perspective (1PP) (Wallace, 2000).

This transition of the science of consciousness into the physical sciences is now entering its fourth successful decade of relief from a long era of explicit-funding-pariah-hood in the physical sciences (Koch, 2019; Seth, 2021). The centuries of prior history of attempts to explain consciousness, in ways too numerous to address here, have been swamped by the knowledge delivered by the 30 years of neuroscience’s active presence in the area. This has occurred despite it being successfully ignored by the bulk of mainstream neuroscientists. In the “correlates-of” paradigm we all set aside the fact that top-down observational correlates do not reveal principled explanations (Seth, 2009). For three decades we have elected to live with that limitation while making impressive progress in exploring for the outward signs of delivery of consciousness by brain activity. Strategically, the “correlates of” paradigm has been a highly effective way to make progress. What it has not done, however, is conclusively isolate the originating principle that might predict brain material’s 1PP.

By its nature, and for good reason, the “correlates-of” paradigm bypasses the true significance of the 1990 event. To see the significance more clearly, consider that producing an abstract “3rd-person-perspective” (3PP) model, that is predictive of observable properties of nature, is the normal, familiar end of the concerns of a physical science. But in 1990 this changed. The ultimate target of the neuroscience of consciousness is an account of “what it is like to be” the studied nature. This is a categorically distinct, novel kind of explanandum. The burden of accounting for the 1PP falls on neuroscience because the human brain’s cranial central excitable cell biology somehow delivers the only instance of a 1PP known to science (this excludes the spinal cord and the peripheral nervous system as originators of subjective experience itself). The human brain’s 1PP is the reason we have a science of consciousness. This is not a “business-as-usual” scientific context. No other physical science has this confluence of circumstances and obligation. For example, no Perovskite geologist is required to account for “what it is like to be Perovskite.” Not so for the neuroscientist and tissue based on excitable cells made of atoms from the same table of elements used in Perovskite.

To deal with this unprecedented explanatory target, the “correlates-of” empirical paradigm was established as an ersatz form of explanation of the 1PP by procedurally rendering it in the familiar, centuries-old 3PP form. It does this in practice by explicitly studying a “correlate of a 1PP *report*.” This is the extra distancing from the target that attracts the “correlates-of” moniker. The 1PP itself *is not observed by the attending scientists*. Instead, the “report” is observed as a highly curated form of hearsay evidence. As a successful empirical method, it has resulted in the bourgeoning and sophisticated knowledge of consciousness that has arisen in the last three decades. However, the intrinsic indirectness and non-uniqueness of the evidence undermines, possibly fatally and indefinitely, the goal of understanding how brains produce consciousness.

If neuroscience is to make a contribution to this rather daunting foundational issue, what might be the form of a solution to the origin of a 1PP? Exactly what is it that brain tissue is “being”? The fundamental physics of electromagnetism (EM) is a very attractive candidate but, effectively, an undiscovered country in the life of the mainstream neuroscientist. Excluding explicit attention to the fundamental physics of the brain has clearly not prevented huge advances in neuroscience but may be precluding investigation of how the brain generates consciousness. In this reframing of approach, what is proposed here isn’t an EM theory of consciousness (EM ToC) but a case for why a ToC should first be sought, by neuroscience, in the EM phenomena of brains.

## Electromagnetism and the Science of Consciousness

The standard model of particle physics is about twice the age of the modern “correlates-of” form of the science of consciousness (Cottingham and Greenwood, 2007; Rich, 2010). In it, physics has already determined what our biosphere and everything in it is made of. It is effectively entirely electromagnetism (electromagnetic fields). This idea applies to anything made of atoms from the table of the elements at a spatiotemporal scale above that of the atomic particles comprising atoms (electrons and nuclei). At the atomic level and above, we and our host environment are defined by three things: space, an EM field system impressed on space (due to subatomic charge and spin content tightly bound up with the subatomic mass), and a gravitational field impressed on space (due to sub-atomic mass, functionally inert in context because it is more than 16 orders of magnitude weaker in force transmission than EM). In rough terms, at the intra-atomic scale, EM fields occupy the space occupied by an atom to the extent of at least 14,999 parts in 15,000. The remaining “1 part” is the interior of electrons and nuclei. When you add in the space between atoms, the proportion of overall spatial occupancy by EM fields is far higher. We humans are nearly entirely EM field objects. In our context of the brain, when we use the words “material” or “physical,” these words (abstractions) refer to EM phenomena.

Therefore, the question “What is it that we are ‘being’?” has an answer in the standard model: “We are ‘being’ EM fields from the atomic level up.” Brevity demands that we avoid going into a discourse on the details, defending it right down into the subatomic intricacies and across the four fundamental force quadrants of the standard model. The standard model’s EM-quadrant/atomic basis of our biosphere is just a basic, well established and proved fact of the physics. More important is how this basic fact impacts a science of consciousness. What is it like to “be” EM fields when the EM fields are configured in the form of a healthy, awake, alert human brain? To be such a configuration of EM fields is, under the right conditions, to be conscious. That is, fundamental physics has already, *prima facie*, determined a bottom-up (fundamental) origin of a 1PP: EM fields. There is literally nothing else there but a functionally irrelevant gravitational field and space. The endogenous EM field expressed by the atomic-level componentry of the brain entirely fills the space occupied by a brain, spilling out from its generating tissue into the surrounding tissue and beyond the skull. An EM ToC merely points out that basic fact and explicitly holds particular aspects of “the brain as an EM field” accountable for a 1PP. As a (bottom-up) claim made with well-established fundamental physics, such a proposal has a clear critical advantage, giving it priority.

What the fundamental physics lacks is an explanation of where EM’s potential for a 1PP comes from, and what specific patterning of brain EM is necessary and sufficient to create a 1PP of a specific kind (qualia or “qualitative feel”) and specific degree (spatial extension, granular resolution, duration, and intensity). Here we set aside this lack as a secondary issue. In terms of a strategic direction for the science, what matters is the obvious centrality of EM fields as the prime candidate for a route to a full explanation of consciousness in fundamental physics terms yet to be formulated. We are all familiar with the EM field system of the brain. Every measurement ever made in support of any ToC involves accessing and characterizing EM properties of the brain (more on this later).

The EM field system impressed on space by brain tissue is therefore not a side effect of cells made of something else. The entire tissue is a single, unitary EM field system impressed on space with atomic-level resolution. For example, there is no special substance that is a neuron. A neuron is a collection of EM fields “behaving neuron-ly” to an observer made of EM fields. “Chemical” or “chemical reaction,” or “chemical pathway” is a reference to EM field activity. “Mechanical” (such as sound propagation/transduction/phonons, or cell deformation) is also an EM phenomenon. “Electro-chemical” is also selecting phenomena entirely comprised of EM. “Quantum mechanics” is not a substance. It is a set of (wave-equation-based) quantizing constraints on EM field expression (such as that determining the electron orbitals in an atom). “Chemical potential” is a population statistic depicting average EM field properties for particular collections of atoms in relation to each other. “Action potentials” are a system of EM field dynamics propagating slowly through space longitudinally following neuronal cell membrane (also an EM field construct). Synapse activity (“electrical” and “chemical”) is an EM field phenomenon. The familiar electrophysiological measurements made in brain tissue detect “total field” in the brain that is a result of the vector-field superposition of myriad individual atomic/molecular field sources that superpose to dominate (spatially, temporally, and in intensity) the underlying atomic/molecular EM field “noise” found at any point in space. “Electrical current” is a transit of an EM field system through space. Ultraweak biophoton and thermal (heat) radiation is also an EM field phenomenon originating in the same system of atomic sources. Diffusion is a collection of randomly colliding atomic EM field systems bouncing off each other due to EM field-based repulsion. To “touch something” with your finger is to engage in an interaction between the EM field system of a finger surface and the EM field of the touched entity.

There is nothing left to describe in a brain that is not EM fields until we get into the interior of the subatomic constituents of atoms. This property is not limited merely to the brain. The pancreas and the heart (or any other organ) are also EM field objects from the atomic level up. What distinguishes the brain’s EM field system from that of any other organ is that its cells can generate an EM configuration conferring the 1PP for humans. Our “Perovskite” rock (above) is also an EM field object, presumably (we conjecture) lacking the specifics of EM field expression that results in a 1PP for the rock.

We can apply the same considerations to previous attempts to explain consciousness using “top-down” abstractions of aggregations of particular formations of EM fields construed as “information,” “signal processing,” “computation,” “thalamocortical loop,” “entropy dynamics,” “resonance,” “reciprocal loops,” “function,” “behavior” and many others. These are all “correlates-of” labels applied to refer to the organization and properties of EM fields. It doesn’t matter whether such depictions of brain tissue operate at molecular/atomic, subcellular, cell organelle, cellular, cell ensemble, cell population, or whole-tissue level. In every case it is EM fields that literally manifest the observable property hypothesized to originate a 1PP. Locating and describing these top-down field-abstractions as “correlates” has, for 30 years, been held up as a route to an explanation of consciousness. But such abstracted “top-down” features that correlate with aspects of consciousness seem to have no explanatory relevance to, or information concerning, the causal basis for having any form of consciousness. An EM ToC seeks an explanation in a separate fundamental physics account of how “being” (bottom-up) EM fields actually originates a 1PP.

These considerations of the state of the science extend even into the long history of EM field theories of consciousness. For interested readers the history and scope of existing EM ToC can be found through reviews (Jones, 2013, 2017; Pockett, 2013). But the details therein are not germane here. In reality all ToC (EM and otherwise) are actually, ultimately, EM field theories sometimes disguised out of view by a chosen kind of abstraction and then empirically supported by measurements also disguising their ultimate EM basis in tissue. We are proposing that we all collectively converge on the reality that it is actually EM fields that originate the 1PP, and engage with fundamental physics in whatever novel manner is necessary to hold it accountable for the origins of a 1PP.

Notice that no existing theory of consciousness is invalidated by this proposal. It is quite possible that one of the plethora of “correlates” is right! This is not contested here. What this article argues is that the “correlate” can be right and yet deliver no actual explanation (no principled account of the origin of the unique explanandum). This is because the EM basis of the correlate is the actual source of the origin of the claimed correlate’s connection to a 1PP.

## Consciousness From Computation

We can further explore the utility of EM in providing explanation of the origin of consciousness by consideration of ToC that do not posit any role for EM. If there are no features, other than those related to signaling between its constituent cells, that neurons contribute to how the brain works, a parsimonious explanation for consciousness is that it too is the result of signal processing (a specific form of computation). This is entirely consistent with the accumulated evidence from the history of studying the brain, which has reinforced, at coarse and fine scales, that the details of conscious experience are associated with the details of brain activity. As previously noted, the evident truth of this does not provide an explanation of why it is so.

The idea that consciousness arises from processing signals (of the now well-defined and well-understood neuronal forms) would give rise to the phenomenon of a 1PP can be called “strong emergence” (or “magical emergence”) because there is, currently, no reason to hold that such a phenomenon would, should, or could follow from the known properties of the system’s constituents (Bedau, 1997; Chalmers, 2006; O’Connor, 2020). This gap in the explanatory sequence has been discussed for as long as the nature of the mind has been considered (Levine, 1983; Van Gulick, 2018), and has more recently been characterized by David Chalmers as the “hard problem”: “*Why should physical processing give rise to a rich inner life at all? It seems objectively unreasonable that it should, and yet it does*” (Chalmers, 1995, 1996, 1997).

The computational view renders consciousness either a rather unimportant feature of brain function or a causally inert epiphenomenon inhering in it. If everything the nervous system does is computation, and thus computation does everything, then there would seem to be no need for consciousness. This disconnects the computational or symbolic representation of brain operation from the physics of the system it represents (the EM physics of nervous system signaling). In other science disciplines, digital models or simulations are used to represent the known and hypothesized attributes and relationships between the elements of a system. Sufficiently accurate and comprehensive models (of the 3PP kind discussed earlier) allow prediction that corresponds to the performance/properties of the real systems (the ones being modeled) and can validate assumptions and hypotheses used in the model.

In the brain sciences, however, the models take on a very different expectation: the simulations are doing exactly the same transformations of signals and data that they are representing in the real system, so must display all phenomena of the system being represented. For example, a computer model of combustion might indicate, numerically, how much heat is produced, but it doesn’t get hot - the simulation uses abstract representations, not the actual physics of combustion. Neuroscientists are entitled to ask what goes missing, in the sense of the heat in the combustion example, when the physics of brain signaling is thrown out and replaced by the physics of a computer. Is the computer and its model really contacting *all* brain phenomena? If there is something missing, how would we know? What procedure might we use to find out? This is the challenge posed by the McCulloch quote (McCulloch, 1965) at the start of this article.

In brain sciences that study consciousness within the paradigm of computation, there is no perceived need to relate the model’s results to the actual physics of brains and neurons. In the current neuroscience paradigm, the physics of brain cells can be entirely ignored once we have sufficient data on activations and connectivity to accurately mimic the signal processing apparently performed by brain signaling physics. This “abstracting away” of the underlying fundamental physics implies that consciousness will emerge from the analogous (or informationally equivalent) simulation of a model of the signal processing that happens in brains. One of the difficulties with accepting this kind of strong emergence as an explanation for consciousness is that it is unclear how to proceed from this position to a deeper understanding of how this happens. “Emergent” as an explanation has the same value as using the term “happens” (Kelly, 1994). Our normal expectation of explanations has been classified as “weak emergence” because they say something about *why* things happen in terms of a mechanistic link between the attributes and actions of the relevant parts and the phenomena they generate. This contrasts with strong emergence, which is literally defined as a form of explanatory failure (Bedau, 1997; Chalmers, 2006; O’Connor, 2020). If consciousness is properly explained, then it would be transformed into weak emergence: a predictable whole resulting from the understood properties of its parts.

But whether the tendency of this “hard problem” to elicit a reliance on strong emergence is seen as a fundamental, or large, or illusory, obstacle to understanding consciousness, it has little bearing on the research being undertaken by neuroscientists working in the standard, albeit tacit, mode of scientific investigation: obtaining 3PP descriptions of nervous system structure and function. Neuroscientists may well suspect that consciousness emerges (somehow) at some higher level of organization to the level of explanation they are pursuing, so a solution is not required of them. This exonerates neuroscientists for being unconcerned about their discipline’s ignorance of consciousness, and absolves them from exploring why EM ToC might offer plausible explanations. We pose that EM should not be ignored because it is actually at the heart of all phenomena in the nervous system, and when neuroscientists measure brain phenomena, the action potentials measured as transmembrane voltage, the “local field potential” (LFP), EEG, and MEG, and so forth, are all aspects of the fine-scale EM phenomena that actually underline the brain’s signaling systems, our characterization of them, and our stimulation of them when we intervene in brain function. To ignore explicit attention to EM, by subsuming it into simplified measurements applied to an abstraction of it, is to cast an irreversible pall of strong emergence over the explanatory discourse of the science of consciousness.

In recent times it has become possible to see EM field interactions within tissue having a direct effect on neuronal excitation. This new signaling mechanism, “ephaptic transmission” shows the causal power of the brain’s endogenous EM fields on its own neural activation. For example, the EM fields associated with neural activity have been shown to generate traveling waves of neuronal excitation in hippocampal pyramidal neurons (Chiang et al., 2019). This characterization of ephaptic transmission in the hippocampus is significant as it implies that EM field propagation can traverse considerable distances in laminated (spatially coherent) and synchronized (temporally coherent) neuronal assemblies - and laminated neuronal assemblies are a fundamental architectural principle across the central nervous system (CNS). This real example of EM fields having a direct effect on neural signaling reveals another advantage inherent in an EM field approach: the provision of a fundamental causal mechanism (via the Lorentz force) within brain signaling. It means that EM ToC offer a plausible physics mechanism linking consciousness to brain causality.

However, even with advantages like this, and like other ToC that identify the informational aspects of neuronal circuits as correlates of consciousness, structures of CNS EM, as an explanation of the origins of consciousness, similarly leaves an explanatory gap (where magical emergence comes in) for how consciousness is generated by EM phenomena. But EM has an aspect that gives it an explanatory future otherwise apparently lacking. Unlike computational ToC, an EM ToC is grounded in the fundamental physics of brain activity.

Even without any claims about which aspects or scales of EM might be relevant for how EM phenomena generate consciousness, it is clear that an EM ToC introduces a significant adjustment to ideas of “substrate independence.” An EM ToC claims that consciousness is substrate dependant. Only a substrate of EM fields of the kind expressed by the brain will do the job. Contrast this with a general-purpose computer running software of any kind. The EM field system physics that is a general-purpose computer need have no direct relationship with the EM field system physics of the brain. In the case of the general-purpose computer (regardless of whether it is claimed conscious or not) the EM fields comprising the general-purpose computer can be organized in any way that is consistent with the execution of the software it hosts (from an abacus to a steam computer). The EM basis of the substrate of a general-purpose computer is radically and irreconcilably different to that of the brain. Note that an (inorganic) artificially originated consciousness based on an EM ToC, for example, requires chip components that generate the same EM phenomena that brain cells generate – at the same spatial and temporal scales. That kind of physics replication activity is, so far, completely missing from the set of options used by neuroscience. It would operate with the same EM field substrate as the natural (organic) brain. The interesting potential future that this suggests is one where the equivalence of a brain and a general-purpose computer can be conclusively scientifically tested based on the idea of substrate *dependence* introduced by an EM ToC.

## Other Ways of Getting Consciousness Into Computations

If strong emergence is not considered to be a satisfactory explanation of how consciousness arises, then a reasonable alternative might be that, rather than it emerging at some point in a complex system, it was actually present all along – perhaps even as an exotic field or particle or similar component that comprises the fundamental fabric of the universe. Some ToC include a proposal that consciousness in some most elemental or fundamental form, is a currently unrecognized (in that it is missing from the standard model of particle physics) basic constituent of the universe. For example Benjamin Libet’s “conscious mental field (CMF)” that “*would not be in any category of known physical fields, such as electromagnetic, gravitational, etc.*” (Libet, 1994). Such proposals recognize that in a more comprehensive appreciation of the nature of the universe’s most basic composition we would appreciate consciousness in the same way that we appreciate that the fundamental constituents of the universe we know about have properties such as mass and spin and charge. Variations of this idea either propose that everything is, to some degree, consciousness [panpsychism (Skrbina, 2007; Goff et al., 2018)] or that consciousness emerges in a recognizable form, or reaches a critical threshold, only under certain constructions. Clearly, brains would be one such construction (indeed currently the only such construction known to us), but even then, there needs to be an explanation of why some aspects of nervous system function have consciousness and why some have not.

The Integrated Information Theory (IIT) ToC, another member of this class of ToC, seeks to find an informational criterion (such as the extent to which information is integrated) to define the presence or the amount of consciousness that certain constructions (biological or otherwise) will possess (Balduzzi and Tononi, 2008; Tononi, 2008; Oizumi et al., 2014; Tononi et al., 2016). To ground the information transformations in consciousness, it has been proposed that all information carries with it, or inherits, or is formed from, a most basic and indivisible mote of consciousness, which is, again, implicitly posed as an undiscovered member or property of an upgraded standard model of particle physics (although it is not presented in standard-model terms). The desire to bring information into the fold of fundamental physics is a topic of exploration within physics more broadly (Walker et al., 2017). We note in passing that an interesting connection between IIT and EM fields has been posed twice to date (Barrett, 2014; McFadden, 2020). This may offer IIT a future as an EM field ToC.

Rather than start a ToC that implicitly relies on an undiscovered fundamental entity and engage in implementing whatever radical changes to the standard model are necessitated by it, EM ToC start with and are located within the relevant quadrant of the existing standard model of particle physics. We already know standard-model EM field properties naturally satisfy the necessary basic requirements of an originator of consciousness. The EM fields are large in spatial extent: the electric and magnetic fields of the brain pervade the entire space occupied by the brain, extending out into the space outside it. The EM fields are impressed on space in exquisite detail consistent with the detail we experience in perceptual fields (such as vision). The EM fields originate at the scale of the membrane in thousands of square meters of a huge electric field spanning the 5 nm membrane enclosure of all neurons and astrocytes. This forms the basis of (a kind of blank canvas for) a nested dynamic hierarchical organizational EM field structure with seven or eight orders of magnitude of spatial detail, extending to the cm scale. The endogenous EM fields of the brain are intense in that they dominate, in a signal strength sense, all the underlying chemical “EM noise” produced by the atomic-level structures generating it in its total form (on a scale that systematically influences its own neuronal excitability – see the above notes on ephaptic transmission).

The EM fields are intrinsically unified: the electric and magnetic fields of the brain are each a single object and inexorably present and modulated when any neural activity occurs. This unification provides a natural route to a solution to another well-known but unexplained property of consciousness: it’s striking and seamless unification of all the experiential modes of vision, audition, touch, olfaction and gustation, along with all the emotions (Cleeremans and Frith, 2003; Bayne, 2010). Natural field superposition also solves the “combination” problem where emergent “wholes,” of a qualitatively unique character can be traced back to its vectorially superadded EM field parts. It facilitates the transformation to weak emergence discussed above. Contents of consciousness delivered by EM fields can enter consciousness merely through the seamless natural integrative superposition (a vector field property) of new field contributions produced by the underlying neural activity originating it. The EM field is also a parsimonious solution to problems related to time, for example, the need for a mechanism that explains how contents of consciousness delivered by EM fields can arrive and leave at the temporal rate and temporal resolution we observe and with the temporal continuity and discontinuity we observe. EM fields have the potential to provide that mechanism. We have already addressed the issue of causality that EM fields uniquely address in well-known physics terms. We will shortly discuss how EM fields naturally possess a potential to address the “symbol grounding/binding” issue. These issues have a long history of prominence in the science of consciousness (Harnad, 1990; Treisman, 1996; Revonsuo and Newman, 1999; Roskies, 1999; Singer, 2001; Chalmers, 2016; Kent and Wittmann, 2021), and EM fields seem naturally suited to potentially offer a solution to them. At least, there is no aspect of these phenomena that seems obviously beyond the scope of EM fields.

However, despite the suitability of EM to potentially account for longstanding, nuanced and unexplained aspects of consciousness, yet again we arrive at the fact that the thing that is missing from an EM field account of consciousness is the troublesome aspect of its delivery: by “being the EM fields.” But this, we hold, is actually *our problem*, not a problem for nature. We are the ones that have failed to bring a 1st-person perspective into fundamental physics. The existing standard model of particle physics is empty of all content specifying “what it is like to be” any of the multitude of standard model entities (of “being” a muon or a neutrino or an EM field, for example). The neuroscience of consciousness, and its novel explanandum, have proved (albeit inadvertently) that the EM fields, a standard model entity, can originate a 1PP. Perhaps this deep and persistent evidence anomaly will motivate some attention by physicists to its standard model. It seems that one way or another, the standard model is up for an eventual makeover to formally introduce the 1PP to its otherwise prodigious predictive capacities. This reinforces the need for a future neuroscience/physics collaboration in the science of consciousness. Meanwhile, the recommended low hanging fruit of a convergence on EM fields is good preparation for it.

## The Simulation Grounding Problem

As well as respecting the fine structure and function of the nervous system components, EM ToC naturally offers neuroscientists the potential to address the symbol binding problem (an issue brain science inherited by adopting paradigms from computer science). “Grounding,” in the sense of models of brain or cognitive function, can take on different definitions (Harnad, 1990). Grounding addresses the sense in which symbols can be regarded as having a reliable relationship with the external environmental inputs that evoke the symbol (or other symbolic representation, such as the distributed activation states in an artificial neural network) or with the outputs to the external environment. A simple thermostat can be said to be grounded in this sense, but not (panpsychism excepted) in the sense that there is any meaning to its operation other than the interpretation of its input, output, and setpoint values in a more comprehensive context, such as in the humans employing or examining its structure and function. Symbols in more complex information processing contexts can stand for abstracted properties of the information. In these cases the complexity comes about by, for example, processing large quantities of information, combining it with previously acquired information, and being directed by explicit or generic objectives and so forth. This permits the analogy of these complex information processes with cognitive functions. Invariably, these information processing models are implemented on digital computers.

Variability in the definitions of grounding is presumably a large part of why it is claimed that the symbol grounding problem has been solved, hasn’t been (but could be) solved, can’t be solved, or isn’t a problem (Taddeo and Floridi, 2005; Steels et al., 2007; Cubek et al., 2015). When considering how consciousness arises, we recognize that various cognitive processes are associated with very distinct and stable conscious states (experiences). These experiences literally are the symbol that becomes bound to brain events. Not only do our cognitions produce experienced conscious states (e.g., frustration, excitement, thirst, redness, fatigue, boredom, anger and so forth), but we are not the least bit unaware of their meaning – we don’t confuse feeling hungry with feeling short of breath; we are not confused about why these conscious states come and go because we are not observing or witnessing them, we *are* those states. The question of grounding in this context is how does the flow of information from interoceptive and exteroceptive systems give rise to the neural activity that generates these utterly familiar and innately interpretable experiences? Neuroscientists would agree that the brain activity occasioned by those inputs, in interaction with the states of the relevant brain regions when receiving the inputs, would dictate the particular quality of the conscious experience. But neuroscientists would likely be very reluctant to say that such states represented in the brain’s activity are grounded by the fact that the signals arise from (for example) vagal afferents from the viscera – if that was true it would not be possible to evoke sensations by stimulation higher up the pathway, and while direct brain stimulation is a very crude and unrealistic substitute for the precise and intricate patterns of activations that occur physiologically, stimulation of the cerebral cortex in awake people can still give rise to conscious experiences appropriate to the modalities known to be present in those cortical regions (Raccah et al., 2021). Phantom limb pathologies also attest to the centrality of cranial brain matter in originating the kind and degree of experiences, resulting in perceptual “grounding” in symbols applied to externalities that do not exist (Giummarra et al., 2007).

If it is held that the origins of meaning can’t be found in the ambient energies in the environment that construe adequate stimuli for sensors, and it is also granted that the inherent meaningfulness of conscious experiences means they must be considered to be grounded, it could be proposed that only consciousness can ground representations expressed in brain activity. For neuroscientists engaged in an EM ToC, this means the basis of grounding is intrinsically there to be found in the activity of the brain’s signaling physics itself – specifically those aspects of its function that are not those abstracted as the signals for information processes in computational models. This makes a sharp distinction between EM ToC and computational theories: the former claims that the crucial fundamental physics mechanisms are the very phenomena that computational theories discard as irrelevant.

## Conclusion

What is proposed here isn’t an EM ToC but a case for why a ToC should be sought in the EM phenomena of brains. It proposes EM as the answer to the challenge: “*Which electrical property provides the most fruitful explanatory basis for understanding consciousness remains an open question*” (Wu, 2018). In the process we find that neuroscience mixed with EM physics locates the center of the study of consciousness. Engaging this possibility, for neuroscientists, means bringing an end to a long era of abstracting-away EM phenomena. Neuroscientists will be required to embrace fundamental physics at a new level of complexity. Neuroscience and physics communities, connected in a joint need to resolve a troublesome and novel explanandum, are likely to be required to accommodate each other’s needs. What the standard model of particle physics might look like after this project is completed, we can only guess at.

Why then, would EM ToC offer an incentive for more neuroscientists to engage with consciousness? The primary reason is that EM fields are the fundamental physics of neurons and glia in the brain. It literally manifests the computations, or signal processing, or information processing/integration activities performed by connected ensembles of cells that we know generate a 1st-person perspective. An EM ToC also has built-in, natural routes to solutions to the thorniest issues of consciousness such as time, unity, binding, combination and causality. Most importantly, it provides the possibility of identifying the outward signs of a mechanism in the normal fundamental terms of EM field physics, as opposed to merely describing the correlates of our mental abstractions of it. A focus on an EM field basis for consciousness does not in any way diminish the role of computation in the operation of the nervous system. Nor does it invalidate any other existing theory of consciousness. Computational activity, or aspects of that activity, will define the particulars of conscious experience, but the computations are not what generates consciousness: that is a deeper level of the fundamental signaling physics originating in the activity of the membrane. That signaling is entirely and only an EM field phenomenon.

## Author Contributions

Both authors listed have made a substantial, direct, and intellectual contribution to the work, and approved it for publication.

## Conflict of Interest

The authors declare that the research was conducted in the absence of any commercial or financial relationships that could be construed as a potential conflict of interest.

## Publisher’s Note

All claims expressed in this article are solely those of the authors and do not necessarily represent those of their affiliated organizations, or those of the publisher, the editors and the reviewers. Any product that may be evaluated in this article, or claim that may be made by its manufacturer, is not guaranteed or endorsed by the publisher.

## Footnotes

1 For example, Computational Models of Cognition, Center for Brains Minds + Machines: Summer Course 2018. See https://www.youtube.com/watch?v=TFyAEHk5asY.
